# The impact of psychological capital on mental health among Iranian nurses: considering the mediating role of job burnout

**DOI:** 10.1186/s40064-016-3099-z

**Published:** 2016-08-22

**Authors:** Mehrdad Estiri, Abbas Nargesian, Farinaz Dastpish, Seyed Mahdi Sharifi

**Affiliations:** Nasr Bridge, Faculty of Management, University of Tehran, P.O. Box: 14155-6311, Tehran, 1417963193 Iran

**Keywords:** Psychological capital, Job burnout, Mental health, Nursing administration

## Abstract

**Background:**

The role of nurses in providing high quality healthcare to patients is so important that creating a desirable working environment to enhance their overall performance is unavoidable. This paper aimed to explore the impact of psychological capital on mental health by investigating the mediating effects of job burnout on this relationship.

**Methods and material:**

The data used in this research was obtained via a survey conducted among selected Iranian nurses in public hospitals. In total, 450 questionnaires were distributed and 384 were completed and returned. Collected data was analysed using Structural Equation Modelling (SEM).

**Results:**

Findings showed that there is a significant relationship between psychological capital, job burnout and mental health; also, there is a significant negative relationship between psychological capital and job burnout, and a significant positive relationship between psychological capital and mental health.

**Conclusion:**

The results have several important practical implications for human resource management in Iranian public hospitals. According to the results of this study, reducing job burnout is an important factor in enhancing psychological capital and can positively enhance nurses’ mental health.

## Background

Nursing is a distinctive profession and the largest component of the health care system, playing a major role in providing consistent and high quality care for patients (Nayeri et al. 2005). Iranian nurses, like the nurses of other countries, are the largest group of health care service providers (Laschinger and Fida 2014). The role of nurses in performing high quality healthcare services for patients is considered important and, accordingly, providing a satisfactory work environment will inevitably enhance their performance.

Nurses have been reported Top of Form as the groups who are at the highest risk of job burnout. The results of former studies suggest that burnout is a serious phenomenon amongst nurses. According to one study, 40 % of examined nurses in four countries suffered from burnout (Aiken et al. 2001). Psychological and emotional needs (Laschinger and Fida 2014; Greenglass et al. 2001) as well as job characteristics such as emotional exhaustion, depersonalization, and personal accomplishments (Schaufeli and Enzmann 1998) expose nurses to high risk of burnout.

Burnout not only affects the health of nurses, but is also considered a threat to the health of patients (Halbesleben et al. 2008; Wang et al. 2012). Accordingly, noting the variables contributing to burnout is important, as it can help to make a positive work environment and enhance the psychological health of nurses as well as improve the health of patients. Previous studies have recognized factors like job dissatisfaction (Zangaro and Soeken 2007) and high workload (Laschinger et al. 2011) as leading to burnout. They have also mentioned factors like absenteeism and turnover (Michie and Williams 2003; Kovner et al. 2007; Leiter and Maslach 2009) as consequences of burnout.

In previous research, psychological capital, or PsyCap, the other concept related to the mental health of employees, is introduced as one of the most important factors affecting the mental health of nurses. This factor can also be a deterrent against stressors such as job burnout (Halbesleben et al. 2008; Montgomery et al. 2006).

PsyCap consists of self-efficacy, hope, resilience and optimism; in other words, taken together, these concepts are referred to as PsyCap in social science research (Luthans and Youssef 2004; Luthans et al. 2007) PsyCap, a construct, consists of four component resources representing ‘one’s positive appraisal of circumstances and probability for success based on motivated effort and perseverance’ that can predict goal attainment and performance (Luthans et al. 2007, p. 550). Furthermore, providing such an environment plays an important role in creating a positive psychological atmosphere for nurses. Positive organizational behavior researchers have conducted various studies on the background and achievements of PsyCap (McMurray et al. 2010).

However the impact of PsyCap on the mental health with regard to the mediator role of job burnout, not being stipulated in previous researches, is one of the topics which can describe and interpret the relationship more accurately among nurses. Another important point is the context in which this research has been done; which adds on the importance of the results of this research. The majority of previous research has employed samples drawn from the United States. There has been little research in other international contexts. However, studies have increased in Asian countries such as China, It is imperative, therefore, to expand understanding of the nurses working environments in an Asian Middle East country such as Iran, where government ownership characterizes the dominant healthcare organizations.

Based on what was stated, there are two main goals in this research. Firstly, examining the relationship between PsyCap and mental health among Iranian nurses. Secondly, investigating the mediating role of burnout between PsyCap and mental health of the nurses. In other words, how PsyCap affects nurses’ mental health through burnout.

## Methods

### Design and sample

A nonexperimental, quantitative design was used in a random sample of staff nurses working in public hospitals across Tehran, Iran. In this research a pre-test was conducted among 30 people. Finally, a total 450 surveys were distributed and 412 were completed and returned. 28 surveys were not assessed due to a lack of reliability. The number of evaluated surveys was 384. (25 % men and 75 % women). Their mean age was 37.2 years and their mean experience was 12 years. Around 91.6 % of the respondents were educated to university level or above. LISREL 9.2 program was used for the analysis. This analysis, constructed upon SEM, as conducted in two stages. In the first stage, reliability and structural validity analyses was performed. In the second stage, the procedures to measure the hypotheses were actualized. To that end, the first explanatory factor analysis was made in order to reveal average values, factor loads and standard values of factor groups within themselves. Finally, in order to analyze the factor groups’ relationship to each other, and the structural validity of the model, confirmatory factor analysis was conducted.

### Measures and variables

#### Independent variable: PsyCap

PsyCap was measured using the PCQ-24 questionnaire developed by Luthans et al. (2007). The scale was formed in terms of 24 items and four dimensions including self-efficacy, hope, flexibility and optimism through a 5-point Likert type (5 = strongly agree, 1 = strongly disagree). A pre-test was conducted to measure the structural validity of the PsyCap scale, as pre-test results indicated that all question were adaptive with the factor structure. The reliability level of this scale (Cronbach Alpha) was determined as 0.93.

#### Mediating variable: job burnout (JB)

JB was measured using the twenty-two-item scale, Burnout Inventory (MBI) developed by Maslach and Jackson (1981). The scale was formed through a 5-point Likert type (5 = strongly agree, 1 = strongly disagree). A pre-test was conducted to measure the structural validity of the Job burnout scale, as pre-test results indicated that all question were adaptive with the factor structure. The reliability level of this scale (Cronbach Alpha) was determined as 0.95.

#### Dependent variable: mental health (MH)

MH was measured using the General Health Questionnaire developed by Goldberg and Hillier (1979). The scale was formed in terms of three dimensions including through a 5-point Likert type (5 = strongly agree, 1 = strongly disagree). A pre-test was conducted to measure the structural validity of the MH scale, as pre-test results indicated that all question were adaptive with the factor structure. The reliability level of this scale (Cronbach Alpha) was determined as 0.90.

## Results

Table 1 shows the means, standard deviations, Internal Consistencies and bivariate correlations of all variables in the study. As expected, all Inter correlations was significant.

**Table 1:** Means, standard deviations, internal consistencies, and inter correlations

Variables	M	**SD**	Cronbach Alpha	1	2	3
1. PsyCap	3.70	0.58	(.93)	–		
2. JB	3.71	0.78	(.95)	−.14**	–	
3. MH	3.61	0.58	(.90)	.46**	−.13**	–

Numbers in parentheses are reliability (alpha coefficient)

** 1 % levels, respectively

In SEM, the condition of accepting measurement model analyzed on data set as a whole within the general structure depends on the fact that goodness-of-fit statistics are on expected level. In evaluating model fit there are various fit indices and certain statistical functions possessed by such indices (Bentler 1990; Joreskog and Sorbom 2004). Maximum likelihood estimation methods were used and the input for the analysis was the covariance matrix of the items. The goodness-of-fit of the model was evaluated using four absolute fit indices (Joreskog and Sorbom 1986): χ^2^ goodness-of-fit statistic, Goodness-of-Fit Index (GFI), Adjusted Goodness-of-Fit Index (AGFI) and the Root Mean Square Error of Approximation (RMSEA). The indices used in this research are Chi square statistics (χ^2^), RMSEA, GFI and AGFI. In fit indices measures, RMSEA = 0.08 and χ^2^ ≤ 3 of ideal values indicates a perfect fit. Likewise, if GFI and AGFI values of .9–.95 or greater are considered indicative of acceptable overall fit (Joreskog and Sorbom 2004). In this research, the values pertaining to fit indices have been on acceptable levels (see Table 2).

**Table 2:** Fit of the measurement models

Measurement model	χ^2^	*df*	GFI (>0/90)	AGFI (>0/90)	RMSEA (<0/08)
PsyCap	7	2	0.99	0.95	0.08
JB	4	5	0.99	0.96	0.06
MH	3	4	0.98	0.94	0.07

At the end of analysis Chi square measurement has been found 98.84 and degree of freedom as (*df*) 33 (*x*^2^/*df* = 2.99; *p* < 0.01), obtained results have been RMSEA = .079, GFI = .95 and AGFI = .90.

The findings of PsyCap has a negative and significant effect on JB (*t* = −5.78; *p* < 0.01). The findings of JB has a negative and significant effect on MH (*t* = −2.96; *p* < 0.01). Therefore, it is reasonable to argue that PsyCap is an equally effective on MH too. This fact is indicated via the PsyCap has positive and significant effect on MH as demonstrated by the analysis results. The increase in the PsyCap positively affects his/her MH (*t* = 8.87; *p* < 0.01). According to Table 3, H1, H2 and H3 hypotheses were supported. Subsequent Sobel tests supported the mediating role of role JB in the relationship between PsyCap and MH (*z* = − 2.45; *p* < .01). Hence, it is concluded that, as expected, JB fully mediate the relationships between PsyCap and MH.

**Table 3:** The result of hypotheses

Hypotheses	Standardized coefficient	*t* value	Result
PsyCap → JB	−.32	−5.78	Supported***
JB → MH	−.18	−2.96	Supported***
PsyCap → MH	.60	8.87	Supported***
χ^2^		98.84	
df		33	
χ^2^/*df* (<3)		2.99	
GFI (>0/90)		.95	
AGFI (>0/90)		.90	
RMSEA (<0/08)		.079	

*** *p* < 0.01. *GFI* goodness-of-fit index, *AGFI* adjusted goodness-of-fit index, *RMSEA* root mean square error of approximation

## Discussion

PsyCap and mental health are two highly effective variables in creating a positive nursing job atmosphere. Doing such research is necessary from the perspective of creating a positive work environment to improve the services provided by health-care service employees.

Positive work environment reflects positive emotional feelings of nurses towards their job. It also can show the cognitive evaluation of their job as well (Peng et al. 2013).

In other words, by positive assessment of their work environment, nurses can show happier and healthier attendance in their work place and other aspects of their lives which leads to mental health probable amplification. In this case, nurses experience higher PsyCap and less burn out. They also may have higher mental health.

In the present study, nurses are selected from a pool of employees in health and medical care owing to the significance of their role in providing public health services to the community and the importance of their job variables. This study tried to clarify the relationship between PsyCap and mental health among nurses, as well as the mediating role of job burnout. The result of this study was generally consistent with those of previous studies. According to the results of this research, PsyCap can negatively affect job burnout. This finding is in accordance with the results of previous studies (Peng et al. 2013; Panatik et al. 2012; de Jonge et al. 2000; Halbesleben et al. 2008; Greenglass et al. 2001).

Based on the positive model of organizational behavior, PsyCap is one of the individual oriented aspects that effectively reduce burnout among nurses by increasing employee occupational happiness. Considering that job burnout is a common and serious phenomenon in nursing (Laschinger et al. 2011), as an important human resource, PsyCap undoubtedly has had a profound effect in on reducing job burnout.

The four components of PsyCap are the basis for job burnout analysis. Nurses with greater self-efficacy are able to show greater readiness in accepting new roles and experiences (Peng et al. 2013; Evers et al. 2002). Optimistic nurses are less vulnerable and more capable of facing job stress and burnout because they think less about the negative aspects of their job. (Peng et al. 2013; Riolli and Savicki 2003).

Furthermore, nurses with a higher ability of adapting to harsh working conditions may experience lower rates of job burnout (Peng et al. 2013; Jackson et al. 2007). Hope, as is one of the components of PsyCap, an have a deterrent effect on burnout. According to the results of various studies, nurses with more hope experience less burnout by setting accurate work objectives and formulate practical action plans in order to succeed (Peng et al. 2013; Snyder 1995).

Undoubtedly, when all of the four components of PsyCap get improved together, that leads to one’s positive evaluation of work condition and success possibility which can increase individual skills in confronting stressful situation,the root of burn out.

The Malleability of Psychological Capital both theoretically and practically has been approved (Peterson et al. 2011) that means the four elements of PsyCap can get developed; PsyCap of nurses, as an illustration, may be enhanced by feedbacks from their peers and mangers or by job redesigning.

According to this study, due to the importance of nurses PsyCap, cultivation of its components would ameliorate nurses’ skills in coping with stressful situations which results in burn out abatement.

Based on the current research findings, the negative effects of burnout on the mental health of nurses have been confirmed. In other words, there is a negative correlation between mental health of nurses and burnout. These findings prove a negative correlation between the mental health and job burnout of Iranian nurses (Abdi et al. 2007; Najafi et al. 2000; Poorkiyani et al. 2012), as well as those from other countries (Peterson et al. 2008; Laschinger and Fida 2014). The effect of job burnout on the mental health of nurses can be explained by factors such as stress, anxiety and depression. Job burnout is a threat to the mental health of nurses through increased stress and anxiety, as well as an increased risk of depression. (Peterson et al. 2008).

The current research findings can ease the better understanding of the importance of nurses’ burn out decreasing plans. Accordingly, hospital managers should consider the plans which increase work passion among nurses and also should provide healthy and happy work environment to obviate negative effects of burn out.

The significant correlation between job burnout and PsyCap on the one hand and burnout and the mental health of nurses on the other has been confirmed in previous research studies.

In this study, the focus was on the mediator role of job burnout in the relationship between PsyCap and the mental health of nurses by considering mentioned correlations. The findings of the present study have demonstrated that nurses who made positive work environment based on the four components of psychological capital are less susceptible to the negative effects associated with bad working conditions such as job burnout.

Furthermore, the decrease in job burnout has a significant and positive correlation with mental health. One of the causes of the decrease in the mental health of nurses is the reduction in anxiety and stressor factors caused by different aspects of the nursing profession which are associated with reducing the impact of job burnout.

According to what has been said, it can be inferred that nurses’ PsyCap not only has positive influence on nurses’ effective skills which has an impact on job burnout, but also can improve their mental health.

## Conclusion

This study is based on a conceptual model that is one of the first to specifically survey the relationship between PsyCap and the mental health of nurses, with regards to job burnout among Iranian nurses. Results of this research represent several important practical implications for nursing administration in Iranian hospitals. Indeed, the study adds to current knowledge concerning the relationship between PsyCap and job burnout, as well as its effect on nurses’ mental health.

The findings confirm previous literature showing that PsyCap has a positive impact on nurses’ mental health, and that job burnout negatively affects it. These results suggest that reducing job burnout as a meaningful factor in enhancing of PsyCap can positively enhance nurses’ mental health.

As in any research, there are some limitations that should be taken into account in the interpretation of these findings. First, the environment of this study may limit its findings; future research may be able to offer new findings in new and different contexts. Second, the instruments used to measure the variables limit the results of this study, though the validity and reliability of the instruments have been verified and they are tenable. However, in future studies, deeper research may lead to the adoption of new standards and represent a new approach to this topic. Finally, all data was based on self-reports, which may introduce bias.
